# Peritoneal dialysis-associated peritonitis caused by *Ureaplasma urealyticum*: A case report

**DOI:** 10.1097/MD.0000000000050996

**Published:** 2026-10-09

**Authors:** Ting Hu, Jinjin Zhang, Geping Yu

**Affiliations:** a Department of Nephrology, Hangzhou First People’s Hospital Tonglu Campus, Hangzhou, Zhejiang, China.

**Keywords:** case report, peritoneal dialysis, peritonitis, targeted next-generation sequencing, *Ureaplasma urealyticum*

## Abstract

**Rationale:**

*Ureaplasma urealyticum* is a rare cause of peritoneal dialysis-associated peritonitis (PDAP) that may be missed by routine culture. Here, we report a culture-negative case in which targeted next-generation sequencing (tNGS) supported rapid pathogen identification and treatment adjustment.

**Patient concerns:**

A 41-year-old woman receiving continuous ambulatory peritoneal dialysis for >10 years presented with acute abdominal pain, cloudy dialysate, fever, and neutrophilic dialysate leukocytosis. The patient was diagnosed with PDAP.

**Diagnoses:**

Repeated bacterial and fungal cultures and acid-fast staining were negative. tNGS of peritoneal dialysate detected *U urealyticum*, which is considered the most likely causative pathogen.

**Interventions:**

Empirical vancomycin plus ceftazidime, followed by vancomycin plus meropenem, was ineffective. The treatment was changed to oral doxycycline plus intraperitoneal levofloxacin after the tNGS results became available. A vaginal *U urealyticum* isolate was subsequently resistant to levofloxacin; therefore, levofloxacin was replaced with intravenous azithromycin.

**Outcomes:**

The abdominal pain resolved, the dialysate cleared, inflammatory markers decreased, the PD catheter was retained, and no recurrence occurred during the 1-month follow-up period.

**Lessons:**

In persistent culture-negative PDAP that does not respond to cell wall-active antibiotics, clinicians should consider cell wall-deficient organisms such as *Ureaplasma*. tNGS may complement conventional microbiological testing, but the results should be interpreted in the clinical context and, when possible, confirmed by an independent method.

## 1. Introduction

Culture-negative episodes account for a clinically important proportion of peritoneal dialysis-associated peritonitis (PDAP) cases and can delay pathogen-directed therapy. *Ureaplasma urealyticum*, a cell wall-deficient organism that requires specialized diagnostic methods, is a very rare cause of PDAP. Here, we describe a patient with persistent culture-negative PDAP in whom targeted next-generation sequencing (tNGS) of peritoneal dialysate detected *U urealyticum* and prompted a change to active antimicrobial therapy. We also summarize previously reported cases to highlight the practical diagnostic and therapeutic considerations.

## 2. Case presentation

A 41-year-old woman was diagnosed with focal proliferative sclerosing IgA nephropathy by renal biopsy in 2014. The disease progressed to end-stage kidney disease in February 2015, and a peritoneal dialysis catheter was inserted. She subsequently underwent continuous ambulatory peritoneal dialysis for >10 years without a previous episode of PDAP. At presentation, there were 4 daily 2000-mL exchanges: 1.5% glucose dialysate, 2.5% glucose dialysate, amino acid dialysate, and icodextrin dialysate. On October 12, 2025, she developed acute abdominal pain, cloudy dialysate, and low-grade fever (maximum, 38.0°C). The dialysate white blood cell count (WBC) count was 400 × 10^6^/L with 90% neutrophils. PDAP was diagnosed, and the patient was admitted for treatment.

On admission, her temperature was 37.7°C, pulse was 97 beats/min, and blood pressure was 97/63 mm Hg. The catheter exit site and the tunnel showed no erythema, swelling, or drainage. Diffuse abdominal and rebound tenderness were observed. The peripheral WBC count was 9.1 × 10^9^/L with 80.4% neutrophils, high-sensitivity C-reactive protein was 120.55 mg/L, and procalcitonin was 1.90 ng/mL. Serum total protein was 64.4 g/L, albumin 35.3 g/L, urea 19.02 mmol/L, creatinine 769.7 μmol/L, and uric acid 236.0 μmol/L.

Before antibiotic administration, 10 mL of cloudy effluent from the 1st drainage bag was aseptically collected, inoculated into aerobic and facultative anaerobic blood culture bottles, and immediately sent to the laboratory. Due to fever, elevated inflammatory markers, and low blood pressure, empirical therapy was initiated with intravenous (IV) and intraperitoneal (IP) vancomycin 500 mg once daily, IV ceftazidime 1 g once daily plus IP ceftazidime 250 mg 4 times daily, and oral fluconazole 150 mg every other day as antifungal prophylaxis. After 3 days, abdominal pain persisted, and the levels of inflammatory markers increased. Repeated dialysate and fungal cultures and acid-fast staining were performed. The regimen was changed to IV and IP vancomycin 500 mg once daily, plus IV and IP meropenem 500 mg every 12 hours. Fluconazole was changed to IV 150 mg once daily and IP 50 mg once daily.

On hospital day 5, the symptoms persisted, and the dialysate WBC count was 2322 × 10^6^/L. Repeated bacterial and fungal cultures and acid-fast staining results were negative. Contrast-enhanced abdominal computed tomography revealed no surgically treatable intraabdominal condition. On October 16, 2025, 5 mL of the peritoneal dialysate was subjected to tNGS. Total nucleic acids were extracted, and libraries were prepared using the Dian Diagnostics ultra-multiplex targeted-amplification panel covering 354 predefined pathogen targets. Sequencing was performed on an MGI platform (MGI Tech Co., Ltd.). The run generated 388,947 effective reads for the analyzed sample. The extraction and amplification controls were also included. The prespecified acceptance criteria required a Q30 score > 85% and detection of internal amplification controls within the expected ranges. Low-quality human reads were removed, and nonhuman reads were aligned to the dian-curated pathogen database by integrating National Center for Biotechnology Information GenBank, NCBI nonredundant comprehensive nucleotide database, and FDA dAtabase for Regulatory Grade micrObial Sequences (accessed on October 17, 2025). A species was classified as positive if all the following thresholds were met: reads per million (RPM) ≥20; the ratio of the sample’s RPM to the maximum RPM detected among all samples in the same batch exceeding 0.05%; detection of specific amplicons from >1 primer pair targeting at least 1 core diagnostic region (>1|1); and the relative RPM (rRPM, sample RPM/no-template control RPM) ≥ 10. *U urealyticum* was detected in 186,496 normalized reads, corresponding to an estimated load of approximately 10^4^ copies/mL. On October 17, the previous antibiotics were discontinued, and the treatment was changed to IP levofloxacin 500 mg loading dose, followed by 50 mg 4 times daily plus oral doxycycline 100 mg twice daily. In accordance with the 2022 International Society for Peritoneal Dialysis guidelines, antifungal prophylaxis was co-prescribed while the patient received an antibiotic course. Therefore, oral fluconazole 150 mg every alternate day was continued. The dialysate WBC count decreased to 847 × 10^6^/L. Vaginal mycoplasma culture showed *U urealyticum*, and susceptibility testing of this vaginal isolate showed levofloxacin resistance. Because no peritoneal isolate was cultured, the strain identity between genital and peritoneal organisms could not be established. On October 19, levofloxacin was discontinued and replaced with intravenous azithromycin 500 mg once daily, while oral doxycycline 100 mg twice daily was continued. By October 21, the dialysate WBC count had decreased to 80 × 10^6^/L.

After the regimen was adjusted, the abdominal pain resolved, the dialysate became clear, and high-sensitivity C-reactive protein and procalcitonin levels decreased (Table 1). The patient was discharged on October 30 with oral doxycycline (100 mg twice daily for 1 additional week). At 1-month follow-up, the patient was free of recurrent peritonitis. Mycoplasma cultures of the peritoneal dialysate and vaginal secretions were negative.

**Table 1:** Clinical course and treatment of peritoneal dialysis-associated peritonitis.

Date (2025)	Oct 12	Oct 13	Oct 14	Oct 15	Oct 16	Oct 17	Oct 18	Oct 19	Oct 20	Oct 21	Oct 22	Oct 23	Oct 28	Oct 30
PD effluent WBC count (×10^6^/L)	400	569	920	2040	2322	847	436	418	237	80	ND	40	ND	ND
PD effluent neutrophils (%)	90	82	94	85	90	86	75	78	60	Not performed*	ND	Not performed*	ND	ND
Serum CRP (mg/L)	120.55	ND	ND	137.05	ND	ND	ND	ND	122.95	ND	ND	48.85	8.8	ND
Serum PCT (ng/mL)	1.9	ND	ND	3.5	ND	ND	ND	ND	4.97	ND	ND	1.24	0.44	ND
Body temperature (°C)	37.7	37.2	37.1	36.7	37.5	36.9	37.5	37.1	37.4	37.6	37.4	37.5	36.5	36.7
PD effluent appearance	Clear	Slightly turbid	Slightly turbid	Turbid	Turbid	Turbid	Slightly turbid	Slightly turbid	Clear	Clear	Clear	Clear	Clear	Clear

Symptoms	Significant abdominal pain					Partial relief of prior symptoms				Mild abdominal pain			No abdominal pain	
Pathogen testing	Negative PD effluent and blood cultures					tNGS: *U urealyticum*		Vaginal mycoplasma culture: *U urealyticum*						
Antimicrobial regimen	IV: ceftazidime 1 g qd + vancomycin 500 mg qd; IP: ceftazidime 250 mg qid + vancomycin 500 mg qd; PO: fluconazole 150 mg qod			IV: meropenem 500 mg q12h + vancomycin 500 mg qd + fluconazole 150 mg qd; IP: meropenem 500 mg q12h + vancomycin 500 mg qd + fluconazole 50 mg qd		PO: doxycycline 100 mg bid + fluconazole 150 mg qod; IP: levofloxacin 500-mg loading dose, then 50 mg qid		IV: azithromycin 500 mg qd; PO: doxycycline 100 mg bid + fluconazole 150 mg qod					Discharged with PO doxycycline 100 mg bid	

bid = twice daily, CRP = C-reactive protein, IP = intraperitoneal, IV = intravenous, ND = not determined, PCT = procalcitonin, PD = peritoneal dialysis, PO = oral, q12h = every 12 hours, qd = once daily, qid = 4 times daily, qod = every other day, tNGS = targeted next-generation sequencing, WBC = white blood cell.

* The differential cell count was not performed because the effluent volume was insufficient.

## 3. Discussion

*Ureaplasma* species are small, cell wall-deficient bacteria that can colonize the lower genital tract. *Ureaplasma parvum* and *U urealyticum* were the principal human species. As these organisms do not have peptidoglycan cell walls, they cannot be visualized reliably by Gram staining and are intrinsically resistant to antimicrobial agents that target cell wall synthesis. Although genital colonization is common, invasive or extragenital infections are uncommon and are more likely to be clinically important in selected hosts or anatomical settings. *Ureaplasma* species have been associated with nongonococcal urethritis, adverse pregnancy, and neonatal outcomes.^[1–6]^

A search of PubMed and major Chinese databases identified 3 previously published cases of *U urealyticum*-associated PDAP (Table 2).^[7–9]^ The reported patients were women aged 28 to 50 years. One episode followed intrauterine device insertion and was temporally associated with menstruation,^[7]^ another occurred after sexual activity,^[8]^ and no precipitating factor was reported in the 3rd case.^[9]^ In the present patient, recent gynecologic evaluation for increased vaginal discharge and the concurrent vaginal *U urealyticum* isolate makes an ascending route biologically plausible. However, because molecular typing was not performed, the genital and peritoneal organisms could not be assumed to be the same strain.

**Table 2:** Published cases of peritoneal dialysis-associated peritonitis caused by *Ureaplasma urealyticum*.

Case	Sex	Age, yr	Primary kidney disease	PD duration	Potential precipitating factor	Pathogen	Detection method	Urogenital *Ureaplasma* testing	Treatment	Outcome	Reference
1	Female	32	Lupus nephritis	5 yr	IUD insertion; menstruation	*Ureaplasma urealyticum*	16S rRNA gene PCR	*Ureaplasma urealyticum*	IUD removal; PO doxycycline	Catheter removal; transfer to hemodialysis; cured	[7]
2	Female	50	Polycystic kidney disease	5 mo	Sexual activity	*Ureaplasma urealyticum*	Mycoplasma culture	*Ureaplasma urealyticum*	IP erythromycin; PO doxycycline; demeclocycline vaginal suppositories	Cured	[8]
3	Female	28	Lupus nephritis	2 yr	Not reported	*Ureaplasma urealyticum*	16S rRNA gene PCR	Not reported	PO doxycycline	Catheter removal; transfer to hemodialysis; cured	[9]

IP = intraperitoneal, IUD = intrauterine device, PCR = polymerase chain reaction, PDAP = peritoneal dialysis-associated peritonitis, PO = oral.

Diagnostic options for *Ureaplasma* include specialized culture, species-specific polymerase chain reaction, 16S rRNA gene polymerase chain reaction, metagenomic next-generation sequencing, and tNGS. Metagenomic next-generation sequencing sequences nucleic acids without a predefined pathogen list, whereas tNGS enriches a fixed set of targets before sequencing. A targeted approach may improve sensitivity and turnaround time for on-panel organisms in samples with abundant host nucleic acids, but it cannot detect organisms outside the panel, and its performance depends on assay-specific validation and reporting thresholds.^[10–14]^ Thus, tNGS results should be interpreted together with specimen quality, organism abundance, conventional microbiology, and the clinical course. In this case, tNGS provided timely and clinically actionable results after repeated cultures were negative.

Because *U urealyticum* lacks a cell wall, β-lactams and glycopeptides are inactive. Potentially active drug classes include tetracyclines, macrolides, and fluoroquinolones, although susceptibility varies.^[15]^ Doxycycline was used in all 3 previously reported cases.^[7–9]^ In our patient, doxycycline plus IP levofloxacin was initially selected because of the persistent systemic and peritoneal inflammation. Levofloxacin was subsequently replaced with azithromycin after susceptibility testing of the vaginal isolate revealed levofloxacin resistance. The susceptibility result was not obtained from a peritoneal isolate and, therefore, served as indirect, rather than definitive, guidance for the treatment of peritoneal infection. Rapid clinical and laboratory response after active therapy supported the catheter retention.

The principal limitation is the absence of orthogonal confirmation of tNGS findings in peritoneal specimens. The remaining dialysate was exhausted, precluding *Ureaplasma*-specific polymerase chain reaction, repeat sequencing, culture under specialized conditions, or molecular typing. In addition, antimicrobial susceptibility testing was performed on the vaginal isolate; however, strain-level identity across the 2 sites was not established. Furthermore, given that the assay relies on a proprietary 354-target panel, the results and thresholds are assay-specific and lack universal standardization across different tNGS platforms. Because sequencing can detect background microbial DNA, these limitations prevent a definitive proof of causality. Nevertheless, the high *U urealyticum* signal in the inflammatory dialysate, repeated negative conventional cultures, concurrent vaginal isolation, biological plausibility, and response to agents active against *Ureaplasma* collectively support *U urealyticum* as the most likely cause. tNGS should be regarded as a complementary test rather than a replacement for conventional microbiology and clinical correlation.^[12,13]^

## 4. Conclusion

Persistent culture-negative PDAP that fails to respond to β-lactams or glycopeptides should prompt consideration of cell wall-deficient and fastidious organisms. tNGS can accelerate the detection of on-panel pathogens and support timely treatment adjustment. However, causal attribution should remain cautious when independent confirmation is unavailable. Antimicrobial selection should integrate the specimen source, susceptibility data, and the clinical response of the patient.

## 5. Patient perspective

The patient reported that the sudden severe abdominal pain and cloudy dialysate caused considerable anxiety, particularly because she feared that the PD catheter might have to be removed and that she might need to change the dialysis modality. She felt relieved when tNGS provided a possible explanation after routine cultures remained negative. She found the oral doxycycline regimen manageable and valued the ability to retain the catheter and continue home-based PD. During the 1-month follow-up visit, the patient reported no residual symptoms.

## Author contributions

**Data curation:** Ting Hu, Jinjin Zhang.

**Supervision:** Geping Yu.

**Writing – original draft:** Ting Hu, Jinjin Zhang.

**Writing – review & editing:** Geping Yu.
